# COVID-19 and Rheumatoid Arthritis Crosstalk: Emerging Association, Therapeutic Options and Challenges

**DOI:** 10.3390/cells10123291

**Published:** 2021-11-24

**Authors:** Saikat Dewanjee, Ramesh Kandimalla, Rajkumar Singh Kalra, Chandrasekhar Valupadas, Jayalakshmi Vallamkondu, Viswakalyan Kolli, Sarbani Dey Ray, Arubala P. Reddy, P. Hemachandra Reddy

**Affiliations:** 1 Advanced Pharmacognosy Research Laboratory, Department of Pharmaceutical Technology, Jadavpur Unversity, Kolkata 700032, India; saikat.dewanjee@jadavpuruniversity.in; 2 Applied Biology, CSIR-Indian Institute of Technology, Uppal Road, Tarnaka, Hyderabad 50000, India; ramesh.kandimalla@gmail.com; 3 Department of Biochemistry, Kakatiya Medical College, Warangal 506007, India; 4 AIST-INDIA DAILAB, National Institute of Advanced Industrial Science & Technology (AIST), Higashi 1-1-1, Tsukuba 305-8565, Japan; rajkumar-singh@oist.jp; 5 Department of Medicine, Mahatma Gandhi Memorial Hospital, Warangal 506007, India; cvalupa-das@gmail.com; 6 Department of Medicine, Kakatiya Medical College Superspeciality Hospital, Warangal 506007, India; 7 National Institute of Technology, Warangal 506004, India; vlakshmij@gmail.com; 8 Department of Biochemistry, GITAM Institute of Medical Sciences and Research, Visakhapatnam 530045, India; kolli.kalyan@gmail.com; 9 Department of Pharmaceutical Sciences, Assam University, Silchar 788011, India; sarbanideyray09@gmail.com; 10 Nutritional Sciences Department, College of Human Sciences, Texas Tech University, 1301 Akron Ave, Lubbock, TX 79409, USA; arubala.reddy@ttu.edu; 11 Department of Internal Medicine, Texas Tech University Health Sciences Center, Lubbock, TX 79430, USA; 12 Departments of Neurology, School of Medicine, Texas Tech University Health Sciences Center, Lubbock, TX 79430, USA; 13 Public Health Department of Graduate School of Biomedical Sciences, Texas Tech University Health Sciences Center, Lubbock, TX 79430, USA; 14 Department of Speech, Language and Hearing Sciences, School Health Professions, Texas Tech University Health Sciences Center, Lubbock, TX 79430, USA

**Keywords:** ACE, ACE2, anti-rheumatic drugs, COVID-19, cytokine storm, immune response, inflammation, rheumatoid arthritis, SARS-CoV-2, therapeutic options

## Abstract

Hyperactivation of immune responses resulting in excessive release of pro-inflammatory mediators in alveoli/lung structures is the principal pathological feature of coronavirus disease 2019 (COVID-19) caused by severe acute respiratory syndrome coronavirus 2 (SARS-CoV-2). The cytokine hyperactivation in COVID-19 appears to be similar to those seen in rheumatoid arthritis (RA), an autoimmune disease. Emerging evidence conferred the severity and risk of COVID-19 to RA patients. Amid the evidence of musculoskeletal manifestations involving immune-inflammation-dependent mechanisms and cases of arthralgia and/or myalgia in COVID-19, crosstalk between COVID-19 and RA is often debated. The present article sheds light on the pathological crosstalk between COVID-19 and RA, the risk of RA patients in acquiring SARS-CoV-2 infection, and the aspects of SARS-CoV-2 infection in RA development. We also conferred whether RA can exacerbate COVID-19 outcomes based on available clinical readouts. The mechanistic overlapping in immune-inflammatory features in both COVID-19 and RA was discussed. We showed the emerging links of angiotensin-converting enzyme (ACE)-dependent and macrophage-mediated pathways in both diseases. Moreover, a detailed review of immediate challenges and key recommendations for anti-rheumatic drugs in the COVID-19 setting was presented for better clinical monitoring and management of RA patients. Taken together, the present article summarizes available knowledge on the emerging COVID-19 and RA crosstalk and their mechanistic overlaps, challenges, and therapeutic options.

## 1. Introduction

Severe acute respiratory syndrome coronavirus 2 (SARS-CoV-2) infection that caused coronavirus disease 2019 (COVID-19) usually produces a mild to moderate respiratory disease [1]. However, it occasionally leads to severe alveolar disease resulting in shortening of breath, reduced oxygen saturation in blood, and pulmonary infiltration in the lung that can substantially contribute to pulmonary failure [2]. Age, the severity of infection, and the existence of comorbidities are potential risk factors in COVID-19 patients [1,3]. Emerging evidence revealed that SARS-CoV-2 develops a specific type of alveolar disease that is clinically different from other acute respiratory syndromes [2]. Immune hyperactivation and cytokine involvement in alveolar structures have been identified as the key contributors to produce severe lung disease in COVID-19 patients. Rheumatoid arthritis (RA) is a chronic autoimmune disease characterized by synovial inflammation and hyperactivation of T cells. Several pro-inflammatory cytokines act as contributing factors in developing synovial inflammation in RA. The patterns of cytokine and immune activation in COVID-19 patients seem to resemble the RA case. Interestingly, some common therapeutic strategies including cytokine inhibition have been found to be fruitful against both COVID-19 and RA [2]. Thus, a possibility of pathological crosstalk is inevitable between COVID-19 and RA.

In general, there is a close association between viral infection and arthritis with a wide spectrum of symptoms ranging from arthralgia to arthritis. Earlier reports revealed that individuals infected with hepatitis C and several alphaviruses frequently develop prolonged arthritis; however, Parvovirus B19, Hepatitis B, and Rubella viruses frequently cause self-limited arthritis. In contrast, respiratory viruses, such as corona and influenza viruses more frequently can cause arthralgia and/or myalgia. Approximately 15 and 44% of COVID-19 patients present arthralgia and/or myalgia, respectively, during the infective stage [4]. Emerging evidence hypothesized that SARS-CoV-2 infection can attack musculoskeletal systems through immune-inflammation-dependent mechanisms, which may develop inflammatory arthritis during the infective or post-infective stage [3,4,5]. However, little is known about the manifestations or worsening of RA by this infection. Since musculoskeletal manifestations phenotypically resemble RA, it has been attempted to find out the association between COVID-19 and RA. In this article, we reviewed the pathological crosstalk between COVID-19 and RA. In addition, our understanding of the risk of RA patients in acquiring SARS-CoV-2 infection and worsening COVID-19 outcomes was critically discussed on the basis of available clinical readouts. The therapeutic strategies and guidelines were conferred referring to recently published literature. Moreover, critical arguments on therapeutic challenges raised in different case studies were discussed in this review.

## 2. COVID-19 and RA Association

Emerging evidence revealed that respiratory viral infections can increase the risk of autoimmune inflammatory arthritis, such as RA [6]. In addition, infections can flare the disease in patients with inflammatory arthritis [7]. Thus, SARS-CoV-2 infection may potentially contribute to RA development or disease flares. So far, little information is available, and it is too early to predict the direct association between SARS-CoV-2 infection and RA development [8]. However, considering the existing evidence, it can be hypothesized that COVID-19 may play a causative effect in RA development or can worsen RA complications. The respiratory tract has been proposed to be the primary site of SARS-CoV-2 infection; however, skeletal muscle, synovium, and cortical bone are the other possible sites of direct SARS-CoV-2 infection [9]. Various anatomical levels of musculoskeletal abnormalities in muscles, bones, and joints were detected in image analysis of COVID-19 patients [10]. Myalgia has been identified as a major clinical presentation of skeletal muscle manifestations in SARS-CoV-2 infection [11]. It can also stand as an important predictive factor for the severity of SARS-CoV-2 infection [12]. Due to the evolving nature of COVID-19, the mechanistic insight of skeletal muscle manifestations is yet to be clearly interpreted. However, muscle fibre atrophy, sporadic and focal muscle fibre necrosis, and immune cell infiltration have been postulated to be the etiological factors involved in the skeletal muscle injury during coronavirus infection [9]. Systemic activation of pro-inflammatory molecules can potentially contribute to muscle fibre proteolysis and decrease protein synthesis [9]. Muscle injury has been regarded as an important contributor to RA-induced morbidity and mortality [13]. Bone and joint manifestations have a broad spectrum in COVID-19 with common viral arthralgia [14]. It has been reported that about 27% of patients demonstrate joint pain even after recovery from COVID-19 disease [15]. Arthralgia in COVID-19 patients more frequently appears along with myalgia. Arthralgia and reduced bone marrow density (BMD) is also frequent in SARS infection, particularly in the patients undergoing glucocorticoid treatment [9]. Glucocorticoids can also increase the risk of osteonecrosis, osteoporosis, and BMD in COVID-19 patients [14]. SARS-CoV-2 infection generally triggers inflammatory mediators including C-X-C motif chemokine ligand 10 (CXCL10), interleukin (IL)-17, and tumor necrosis factor-alpha (TNF-α), which play causative roles in the initiation of osteoclastogenesis and reduction in osteoblast differentiation and proliferation, resulting in a net fall in BMD [9]. In addition, IL-1β, IL-6, and TNF-α activation during SARS-CoV-2 infection may impart arthralgias or osteoarthritis progression by inducing chondrolysis in an inflammation-dependent mechanism [9]. Activation of IL-1, IL-6, and TNF-α is also implicated in joint degeneration and synovial cell activation in RA pathology.

### 2.1. Can SARS-CoV-2 Infection Trigger RA Development?

In general, viruses can produce arthritis either through direct colonization at the joints or through aberrant immune-inflammation reactions produced during the host response to the infection. Earlier reports mentioned that respiratory viruses can be associated with RA development [7]. Moreover, the evidence of SARS-CoV-2 infection in rheumatic and autoimmune manifestations is not uncommon [16]. The onset of arthritis after SARS-CoV-2 infection has been postulated earlier [17]. Derksen and coworkers reported that 3 out of 61 COVID-19 positive patients developed polyarthritis that resembles regular RA after infection; however, they did not find any increase in anti-citrullinated peptide antibody (ACPA) seroprevalence [18]. Thus, COVID-19-provoked hyperactivation immune-inflammatory response may serve as a potential causative factor in developing RA through a citrullination-independent pathway. In a recent report, Perrot and colleagues first mentioned a case of ACPA-positive RA development immediately after SARS-CoV-2 infection that further worsens RA symptoms [19]. In another report, Roongta and peers reported a case of seropositive RA after SARS-CoV-2 infection, which has been mentioned as the sixth case of COVID-19-induced seropositive RA manifestation. All six patients represented negative serology before infection [20]. Despite a few cases of RA development after COVID-19 had been reported in available clinical readouts, more information is required to know that whether RA manifestation following SARS-CoV-2 infection is connected or is a coincidence.

### 2.2. Can RA Increase the Risk of Acquiring COVID-19 Infection?

Emerging evidence revealed that patients with immune-mediated inflammatory diseases are more vulnerable to represent severe SARS-CoV-2 infections than that of the general population, which may arise due to their immune dysfunction and as a consequence of immunosuppressant therapy [21]. Accumulated data of seven case-control studies showed that the prevalence of symptomatic COVID-19 is almost two times greater in patients with immune-mediated inflammatory diseases compared to the general population [22]. In a large worldwide case series of rheumatoid patients, COVID-19 severity and death were significantly higher in RA patients as compared to the general population [23]. According to the COVID-19 global rheumatology alliance (GRA) registry, out of 7263 COVID-19 cases, 2956 patients were documented to have RA, which is accounting for 40.7% [24]. Other studies are in agreement with these observations and revealed that RA patients are more susceptible to acquiring SARS-CoV-2 infection [25]. Immunosuppressant therapy has been regarded to be considerably associated with the risk of SARS-CoV-2 infection [22]. In contrast, a Euro-COVIMID cross-sectional study interpreted that neither immune-mediated inflammatory diseases nor immunosuppressant therapy could produce any significant change in COVID-19 severity and mortality as compared to the general population [22]. Simmon and colleagues claimed that treatment with anti-cytokine drugs represents a low prevalence of COVID-19 seroconversion in patients with immune-mediated inflammatory diseases [26]. However, this protective mechanism is yet to be clearly deciphered. Considering the majority of clinical readouts, it is reasonable to mention that RA patients are at an increased risk of getting SARS-CoV-2 infection and developing serious illness from COVID-19.

### 2.3. Can RA Worsen COVID-19 Outcomes?

OpenSAFELY, a health analytics platform holding healthcare records of 40% of patients in England revealed that the presence of rheumatic diseases slightly increases the risk of COVID-19 mortality as compared to the patients without these diseases [27]. In a recent UK Biobank cohort analysis, Topless and colleagues revealed that RA is a potential risk factor in COVID-19-related death [28]. RA patients are more likely to present comorbidities like asthma, chronic obstructive pulmonary disease, hypertension, cardiovascular diseases, and diabetes [29]. Thus, RA patients are always at a high risk of COVID-19 severity and death as compared to the patients without RA. RA medication may also contribute to COVID-19 outcomes. In a prospective study involving 103 patients with inflammatory arthritis (RA and spondyloarthritis), Haberman and co-workers conferred that immunosuppressant therapy could worsen COVID-19 outcomes as compared to the patients receiving cytokine inhibitors [30]. Similar observations have been reported by others [7,26]. Moreover, it has also been demonstrated that RA can be worsened by infection through the iatrogenic effect of immunosuppressants [7]. Thus, it could be said that RA represents a potential threat in worsening COVID-19 outcomes and increases the risk of COVID-19-related death and hospitalization.

## 3. Immune-Inflammatory Activities in SARS-COV-2 Infection and RA 

**Figure 1:**
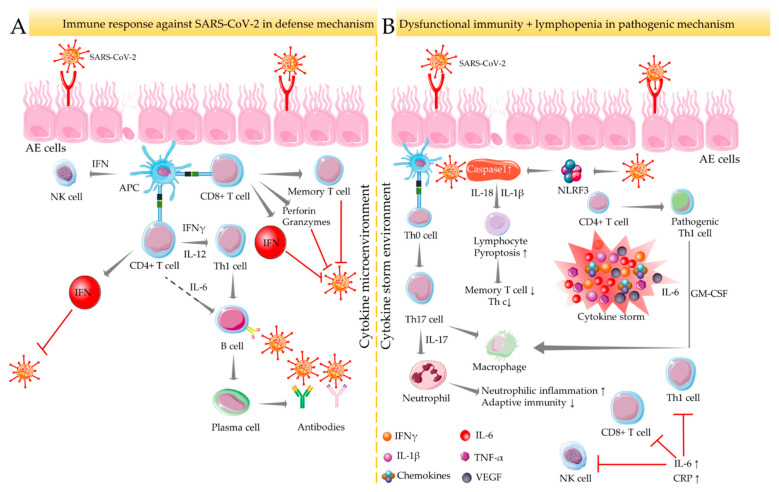
Immune-inflammatory activities in SARS-CoV-2 infection. Immune response against SARS-CoV-2 under normal defense (**A**) and dysfunctional pathogenic (**B**) mechanisms. In normal defense mechanisms against SARS-CoV-2, CD4+ helper T cells contribute to the overall adaptive response by recruiting Th1 cells and endorsing B lymphocyte differentiation to produce specific anti-SARS-CoV-2 antibodies. CD4+ and CD8+ T cells also produce IFN to neutralize SARS-CoV-2. APC endorses T cell-mediated immune responses in the cytokine microenvironment. Dysfunctional immune response coupled with lymphopenia yields severe COVID-19 outcomes by endorsing a hyperinflammatory state referred to as a “cytokine storm.” In a cytokine-enriched environment, SARS-CoV-2 induces pyroptosis of lymphocytes via activating NLRP3 inflammasome and caspase 1, which results in the decline in memory Th and regulatory T cell population. APC activates Th17 cells that endorse neutrophilic inflammation and suppress adaptive immunity by activating neutrophils. This pathogenic pathway is further potentiated via pathogenic Th1 cells’ activation by CD4+ T cells. Arrows indicate the downstream cellular events, and red lines indicate inhibition. “↑” indicates upregulation/activation, and “↓” indicates downregulation/suppression. AE cells: alveolar epithelial cells; APC: antigen-presenting cell; CRP: C reactive protein; GM-CSF: granulocyte-macrophage colony-stimulating factor; IFN: interferon; IL: interleukin; NK cells: natural killer cells; NLRF3: NLR family pyrin domain containing 3; TNF: tumor necrosis factor; VEGF: vascular endothelial growth factor.

Upon SARS-CoV-2 infection, infected host cells rapidly execute both innate and adaptive immune responses, which serve as the initial line of defense against COVID-19 [31] (Figure 1A). CD8+ cytotoxic T lymphocytes can recognize SARS-CoV-2 structural proteins presented by infected epithelial cells and induce apoptosis to virus-targeted cells by releasing proapoptotic factors, such as perforin and granzymes [32]. CD4+ helper T cells contribute to the overall adaptive response by assisting cytotoxic T cells. Upon infection, CD4+ helper T cells recruit T helper (Th)1 cells and endorse differentiation of B lymphocytes into plasma cells, which, in turn, produce specific anti-SARS-CoV-2 antibodies [32]. In addition, CD4+ and CD8+ T cells produce type-I interferon (IFN), which acts together with antiviral antibodies to neutralize SARS-CoV-2 [31] (Figure 1A). However, T cell-mediated immune responses depend on the antigen-presenting cell (APC)-mediated cytokine microenvironment. Dysfunctional immune response in association with lymphopenia causes severe pulmonary and other systemic injuries and potentially yields death by endorsing a hyperinflammatory state mediated through the massive release of cytokines and chemokines [31]. This phenomenon is referred to as a “cytokine storm.” SARS-CoV-2 can trigger caspase 1 activation via upregulating the NLR family pyrin domain containing 3 (NLRP3) inflammasome, which subsequently induces pyroptosis to the lymphocytes through the recruitment of IL-1β and IL-18 [33] (Figure 1B). A significant decrease in the number of memory T helper cells and regulatory T lymphocytes has been reported in COVID-19 patients [31]. The immunological basis of severe COVID-19 pathogenesis could be associated with the development of pathogenic T cell phenotypes and the massive production of proinflammatory mediators. SARS-CoV-2 infection can divert the commitment of CD4+ T lymphocytes towards a pathogenic Th1 cell immunophenotype resulting in the release of pro-inflammatory cytokines, such as IL-6 and granulocyte-macrophage colony-stimulating factor (GM-CSF) [34]. This hyperinflammatory milieu can subsequently endorse the differentiation of monocytes into macrophages or APCs by endorsing IL-6 secretion [34]. An excess of systemic IL-6 endorses C-reactive protein production, impairs immunophysiological function of Th1 cells against SARS-CoV-2, and inhibits physiologic actions of CD8+ T and natural killer (NK) cells [31] (Figure 1B). After penetration into lung tissue, SARS-CoV-2 reaches APCs and endorses differentiation of Th0 cells into Th17 lymphocytes, which subsequently triggers IL-17 production. IL-17 consequently endorses macrophage activation and neutrophil recruitment, which then promotes neutrophilic inflammation and suppresses adaptive immune responses against the virus [31]. Several comorbidities favour this immunopathological swing of Th17/IL-17 hyperactivation in COVID-19 patients [35] (Figure 1B). Emerging evidence revealed that elevated levels of plasma cytokines, such as IL-1β, IL-2, IL-4, IL-6, IL-7, IL-10, IL-17, IL-18, TNF-α, vascular endothelial growth factor (VEGF), granulocyte-macrophage colony-stimulating factor (GM-CSF), IFN-γ, etc. and chemokines (CXCL-8, CXCL-10, CCL-1, CCL-2, CCL-3, CCL-4, etc.) are directly associated with COVID-19 severity [36,37].

**Figure 2:**
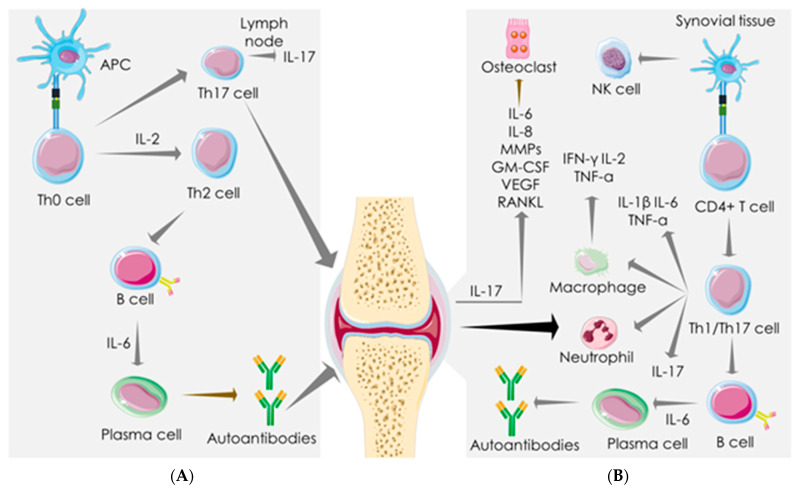
Immune-inflammatory activities in rheumatoid arthritis (RA). Dysfunctional adaptive immunity against “self” antigens and dysregulated cytokine set-ups are the hallmark of RA pathogenesis in the lymph nodes (**A**) and synovial tissue (**B**). APC leads to Th2 and Th17 activation in the lymph node. CD4+ T cells induce chronic autoimmune response and elicit inflammation via endorsing Th1/Th17 axis in synovial tissue. Th1/IL-17 activation elicits pro-inflammatory mediators, activates immune cells, and endorses B cell differentiation to produce autoantibodies in synovial tissue. IL-17 produced by activated Th17 elicits pro-inflammatory cytokines, VEGF, and MMPs in synovial fibroblasts. Arrows represent the downstream cellular events. APC: antigen-presenting cell; GM-CSF: granulocyte-macrophage colony-stimulating factor; IFN: interferon; IL: interleukin; MMP: matrix metalloproteinase; NK cells: natural killer cells; RANKL: receptor activator of nuclear factor kappa-Β ligand; TNF: tumor necrosis factor; VEGF: vascular endothelial growth factor.

Immunologically, RA is characterized by dysfunctional innate immunity, adaptive immunity against “self”-antigens, and dysregulation in cytokine set-ups. CD4+ T cells contribute to the chronic autoimmune response of RA via antigenic activation of naive CD8+ T cells, which, in turn, triggers inflammation via massive production of pro-inflammatory mediators [38] (Figure 2). TNF-α, IL-1β, and IL-6 play crucial roles in stimulating joint inflammation in RA [39]. The role of CD4+ T-cells in inducing chronic inflammation in the joints of RA patients has been exposed [38]. Emerging evidence revealed the predominant role of Th cells in the pathogenesis of RA [39]. Th1 hyperactivation in RA triggers the secretion of pro-inflammatory mediators, such as IFN-γ, IL-2, and TNF-α [40]. In addition, Th1 cells can recruit macrophages to act as APCs. Th2 cells also play critical roles in RA pathogenesis, which promote B lymphocyte differentiation to produce antibodies (IgE) [38]. In addition, the Th17/IL-17 axis plays a central role in RA pathogenesis [38]. IL-17 enhances the production of IL-6, IL-8, VEGF-A, and matrix metalloproteinases (MMP-1 and -3) in RA synovial fibroblasts [38] (Figure 2). The enhanced plasma level of proinflammatory cytokines (IL-1β, IL-2, IL-4, IL-6, IL-7, IL-12, IL-16, IL-17, IL-18, TNF-α, GM-CSF, IFN-γ, etc.) and chemokines (CXCL-8, CXCL-10, CCL-1, CCL-2, CCL-3, CCL-4, CCL-5, etc.) remains as a diagnostic feature in RA patients [41]. Thus, it is reasonable to conclude that the pathophysiology of both SARS-CoV-2 infection and RA share a similar mechanistic pathway of aberrant immune response resulting in hyperactivation of the cytokine–chemokine axis.

## 4. Mechanistic Similarity between SARS-COV-2 Infection and RA 

### 4.1. Angiotensin-Converting Enzyme (ACE)-Dependent Pathway

**Figure 3:**
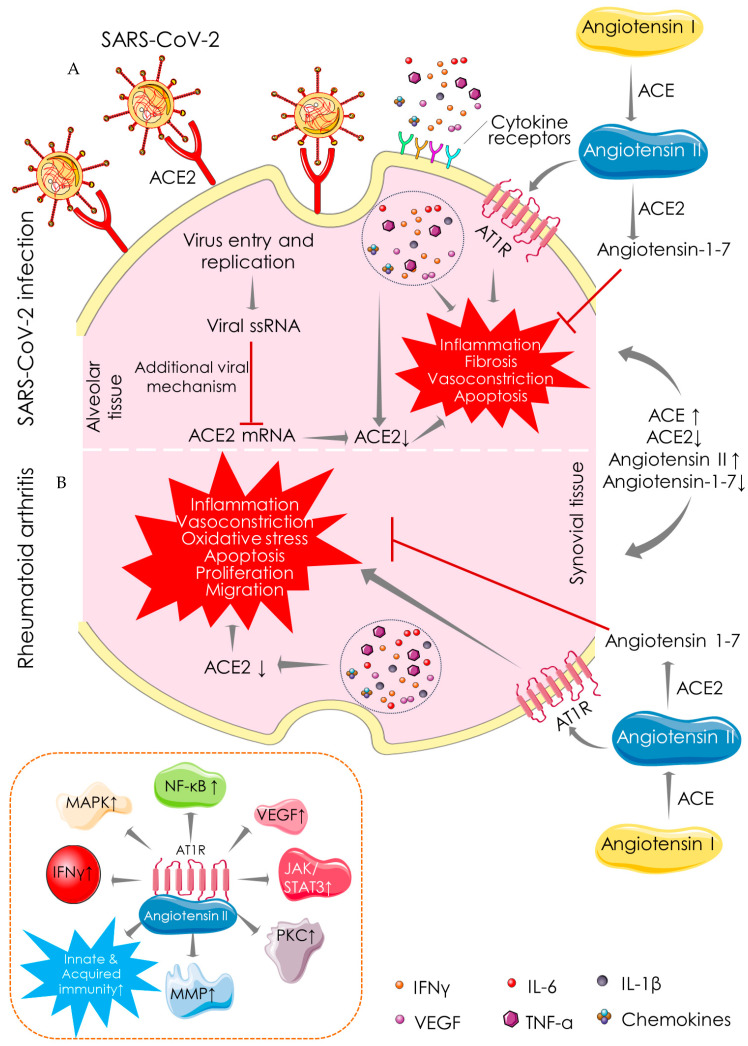
Angiotensin-converting enzyme (ACE)-dependent pathway showing the mechanistic similarity between SARS-CoV-2 infection (**A**) and RA (**B**). ACE catalyses the conversion of angiotensin I to angiotensin II, which is involved in the pathogenesis of both COVID-19 and RA by promoting inflammation, fibrosis, vasoconstriction, and apoptotic activities. In contrast, ACE2 catalyses the conversion of angiotensin II to angiotensin-1-7 and shares identical protective functions in both COVID-19 and RA. Arrows indicate the downstream cellular events, and red lines indicate inhibition. “↑” indicates upregulation/activation, and “↓” indicates downregulation/suppression. ACE: angiotensin-converting enzyme; ACE2: angiotensin-converting enzyme 2; AT1R: angiotensin II receptor type 1; IFN: interferon; IL: interleukin; JAK: janus tyrosine kinase; MAPK: mitogen-activated protein kinase; MMP: matrix metalloproteinase; NF-κB: nuclear factor kappa-light-chain-enhancer of activated B cells; PKC: protein kinase C; STAT: signal transducer and activator of transcription; TNF: tumor necrosis factor; VEGF: vascular endothelial growth factor.

Immune-inflammatory disorders can be associated with ACE/ACE2 imbalance [42]. ACE promotes the conversion of angiotensin I to angiotensin II, while ACE2 catalyzes the conversion of angiotensin II to angiotensin-1–7, which exhibits anti-inflammatory, anti-fibrotic, anti-apoptotic, anti-proliferative, and vasorelaxation effects. ACE2 maintains renin-angiotensin system (Ras) homeostasis to restore normal physiological processes in critical tissues/organs. The role of ACE2 in SARS-CoV-2 infection stands itself as an irony. ACE2 as a receptor serves as a potential cellular target for SARS-CoV-2 to enter the target cells [43] (Figure 3). ACE2 binding is essential for the entry of SARS-CoV-2 into the host cells; however, recent evidence revealed a key role of heparan sulfate in facilitating their interaction and thereby potentiating SARS-CoV-2 cell entry and infection [44,45]. In contrast, ACE2 as an enzyme plays a protective role in SARS-CoV-2 infection [43]. The S-protein of SAR-CoV-2 binds to ACE2, resulting in a suppression of ACE2 expression and promoting COVID-19 pathogenesis [46]. Inhibition of ACE2/angiotensin-1–7 activates rapidly accelerated fibrosarcoma (Raf)/mitogen-activated protein kinase (MAPK) cascade, which in turn shares identical pathological signalling in both COVID-19 and RA [47] (Figure 3). ACE2 activators have been proposed to produce dual benefits in COVID-19 treatment: firstly, by inhibiting the binding of S-protein of SARS-CoV-2 to ACE2 and secondly by offering the protective effect of the ACE2 enzyme [48]. ACE2 activation can also be beneficial in RA, which can mitigate inflammation, vasoconstriction, oxidative stress, apoptosis, proliferation, and migration in synovial tissue (Figure 3). ACE activation promotes the accumulation of angiotensin II, which could be pathologically involved in both COVID-19 and RA. In an inflammatory milieu, angiotensin II is known to trigger inflammatory responses and vascular permeability by enhancing the production of prostaglandins and VEGF [49]. These inflammatory mediators further endorse nuclear factor kappa-light-chain-enhancer of activated B cells’ (NF-κB) activation, which intensifies the inflammatory responses and promotes infiltration of inflammatory cells into damaged tissues [49]. Moreover, angiotensin II can endorse lymphocyte proliferation and activation, as well as the formation of free radicals in leucocytes [50]. ACE inhibitors and angiotensin receptor blockers have been regarded to be beneficial in COVID-19, delaying the binding of SARS-CoV-2 by activating ACE2 and increasing the availability of angiotensin-1–7 [48]. Pharmacological inhibition of ACE and angiotensin II can reduce the risk of mortality in COVID-19 patients [49]. ACE inhibitors limit the production of pro-inflammatory cytokines by suppressing NF-κB activation, and this anti-inflammatory mechanism can be effective against both diseases [51]. ACE inhibitors have been proven to improve vascular endothelial function in RA patients [50]. Thus, it could be said that both COVID-19 and RA share a common mechanistic pathway of immunopathogenesis mediated through aberrant ACE/ACE2 activities.

### 4.2. Macrophage-Mediated Pathway

**Figure 4:**
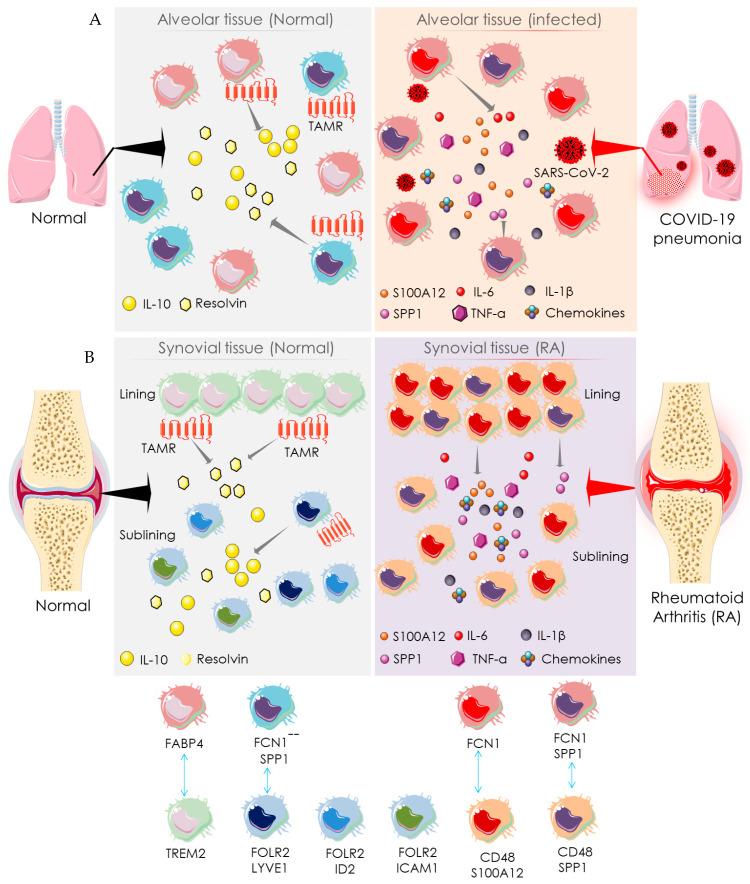
Macrophage-mediated pathway showing the mechanistic similarity between SARS-CoV-2 infection and RA. Schematic diagram showing the pro-inflammatory function of distinct macrophage subsets in the normal (left side) and SARS-CoV-2 infected (right side) alveolar tissue (**A**) and synovial tissue (**B**) of normal (left side) and RA patients (right side). The identity of distinct participating macrophage subsets is shown at the bottom. Arrows indicate the downstream cellular events; double-headed arrows represent the similarity in transcriptomic homology and regulatory activities. “+” indicates positive expression, and “-“ indicates negative expression. FABP4: fatty acid-binding protein 4; FCN1: ficolin-1; FOLR2: folate receptor beta; ICAM1: intercellular adhesion molecule 1; IFN: interferon; IL: interleukin; LYVE1: lymphatic vessel endothelial hyaluronan receptor 1; S100A12: S100 calcium-binding protein A12/calgranulin C; SPP1: secreted phosphoprotein 1/osteopontin; TAMR: TAM receptor; TREM2: triggering receptor expressed on myeloid cells 2.

Macrophages present in bronchial and synovial tissues are heterogeneous (Figure 4). Healthy lungs represent alveolar macrophages expressing fatty-acid-binding protein 4 (FABP4), which help in maintaining gas exchange and compliance. During SARS-CoV-2 infection, the number of FABP4-expressing (FABP4^positive^) alveolar macrophages is substantially reduced, and thus gas exchange is compromised [52] (Figure 4). Bronchoalveolar lavage fluids from COVID-19 patients exhibit more distinct types of macrophages than resident macrophages present in healthy alveoli [53]. The alveolar tissue of COVID-19 patients abundantly presents two distinct macrophage clusters expressing ficolin-1 (FCN1), which can be differentiated by their relative expression of secreted phosphoprotein 1/osteopontin (SPP1) [53]. These are categorized as only FCN expressing macrophages (FCN^positive^) and macrophages that express both FCN and SPP1 (FCN^positive^SPP1^positive^). FCN^positive^ macrophages are probably involved in COVID-19 pathogenesis through a strong adaptive immune response mediated through CD8+ T cells. However, their specific pathological role is yet to be established. Similarly, synovial tissue also represents distinct macrophage subsets in RA patients as compared to healthy people [54] (Figure 4). In healthy joints, macrophage clusters expressing triggering of receptors expressed on myeloid cells 2 (TREM2), such as TREM2^high^ and TREM2^low^ in association with the macrophages expressing both the folate receptor beta (FOLR2) and lymphatic vessel endothelial hyaluronan receptor 1 (LYVE1), constitute synovial tissue lining. The macrophages expressing FOLR2 in association with LYVE1, inhibitor of DNA binding 2 (ID2), or intercellular adhesion molecule 1 (ICAM1) form the synovial sub-lining. These are categorized as FLOR2^positive^LYVE1^positive^, FLOR2^positive^ID2^positive^, and FLOR2^positive^ICAM1^positive^ macrophage clusters (Figure 4). As compared to healthy joints, synovial tissue of RA patients additionally represents two distinct types of macrophage clusters: one is highly expressing CD48 and S100A12 (S100 calcium-binding protein A12/calgranulin C), while another is expressing both CD48 and SPP1 (Figure 4). Both CD48^high^S100A12^positive^ and CD48^positive^SPP1^positive^ macrophage clusters have been revealed to be associated with RA pathogenesis via producing pro-inflammatory mediators such as IL-1β, IL-6, TNF-α, MMPs, and chemokines and inducing pathogenesis to the adjacent stromal tissue [53]. FCN^positive^ and FCN^positive^SPP1^positive^ macrophages in bronchoalveolar lavage fluids from COVID-19 patients share a transcriptional homology with pathogenic CD48^high^S100A12^positive^ and CD48^positive^SPP1^positive^ macrophage clusters in the synovial tissue of RA patients (Figure 4) [53]. In addition, both share similar functional characteristics in the respective tissues [53]. Similarly, FABP4^positive^ alveolar macrophages in the bronchoalveolar lavage fluids from healthy individuals share transcriptional and functional homology with TREM2 expressing synovial macrophages in healthy joints (Figure 4) [53]. Both TREM2^positive^ and FOLR2^positive^LYVE1^positive^ macrophages resolve inflammation by activating anti-inflammatory mediators and repair stromal cells by recruiting Mer receptor tyrosine kinase (MerTK) and its ligand growth arrest-specific protein 6 precursor (GAS6) [53]. MerTK, a member of the TAM family with its ligands GAS6 and vitamin K-dependent protein S (PROS1), contributes to an inflammation-alleviating effect. FABP4^positive^ macrophages express Axl receptor tyrosine kinase (Axl) and PROS1, which aids in reducing COVID-19’s severity. Both FABP4^positive^ and TREM2^positive^ macrophages share a similar functional role of a homeostatic brake on inflammation in alveolar and synovial tissues, respectively (Figure 4). Taken together, the alveolar macrophages in healthy individuals share homologies in transcriptomic profiles and regulatory pathways with the macrophages in synovial tissue of healthy individuals. Similarly, macrophages in the alveolar tissue of COVID-19 patients are homologous to that of the synovial macrophages in RA patients. Thus, both SARS-CoV-2 infection and RA share a common mechanistic pathway of immunopathogenesis driven by the activities of analogous macrophage clusters.

## 5. Therapeutic Management and Challenges

### 5.1. Recommendation for Anti-Rheumatic Drugs in the COVID-19 Setting

Patients with RA generally represent a compromised immune system, which makes them susceptible to SARS-CoV-2 infection [25]. Treatment with immunosuppressant drugs may further increase the risk of acquiring SARS-CoV-2 infection [22]. In addition, clinical features of RA flares and SARS-CoV-2 infection frequently overlap [29]. Both RA- and COVID-19-positive patients represent some common symptoms like arthralgia, myalgia, and other inflammatory disorders. RA-mediated interstitial lung disease often mimics COVID-19 symptoms. Moreover, RA patients frequently represent increasing evidence of comorbidities [55]. Thus, clinical management of RA itself stands as a challenging task in the present COVID-19 setting. Among the possible therapeutic options, the American College of Rheumatology (ACR) and the European League Against Rheumatism (EULAR) recommended several guidelines regarding the use of RA medication in the COVID-19 pandemic [56,57]. Glucocorticoids have been recommended at the lowest possible dose even in COVID-19-positive cases, and sudden withdrawal has been discouraged. Non-steroidal anti-inflammatory drugs (NSAIDs) have been proposed to be continued unless severe COVID-19 outcomes present to multiple organs. Among disease-modifying antirheumatic drugs (DMARDs), conventional synthetic DMARDs (csDMARDs) have been recommended to be continued; however, leflunomide, methotrexate, and sulfasalazine are suggested to be avoided in suspected or confirmed COVID-19 cases. All biological DMARDs (bDMARDs) except for IL-6 inhibitors and all targeted synthetic DMARDs (tsDMARDs) have been advised to be discontinued in suspected or confirmed cases of COVID-19. Regarding re-initiation of DMARDs, ACR recommended restarting these drugs within 7–14 days of symptom resolution or within 10–17 days of the positive report for symptomatic and asymptomatic patients, respectively [55]. However, treatment resumption is required on an individual basis for patients recuperating from a serious illness [56]. Thus, adjustments to medication should be done on an individual basis considering disease severity, and specific attention must be given to the recommendations of using antirheumatic drugs in the COVID-19 setting by different professional rheumatology associations, such as the ACR and the EULAR.

### 5.2. Challenges with Anti-Rheumatic Agents in the COVID-19 Setting 

Despite this, no direct association has been established between specific RA medication and the COVID-19 development or outcomes, and some reports claimed that RA patients exhibit an increased risk of serious infections (Table 1). Accumulating evidence showed the protective role of glucocorticoids in COVID-19, which is predominantly mediated through their immunosuppressive effects to overturn hyperinflammatory states in the late phases of SARS-CoV-2 infection [29]. The World Health Organization (WHO) recommended glucocorticoid treatment in severe/critical COVID-19 cases. In contrast, Russell and colleagues claimed that glucocorticoids are not clinically effective against COVID-19 lung injury [58]. Moreover, a report claimed that a moderate-to-high dose of glucocorticoids can increase the risk of poor outcomes [59]. In a small cohort, Haberman and colleagues also found that glucocorticoid treatment worsens COVID-19 outcomes in patients with inflammatory arthritis [30]. According to the Global Rheumatology Alliance and other reports, glucocorticoids (≥10 mg/day) are associated with an increased rate of hospitalization for COVID-19 in patients with rheumatic diseases [23]. Glucocorticoids at high doses have been regarded as a potential risk factor for COVID-19 patients representing rheumatic diseases [60]. RA patients chronically receiving glucocorticoids have been advised to avoid abrupt withdrawal of the drugs even after acquiring SARS-CoV-2 infection and to continue glucocorticoid treatment with the lowest possible doses [25]. Thus, the dose of a glucocorticoid stands critical in this aspect.

Some reports claimed that the use of NSAIDs does not produce any serious adverse manifestation in COVID-19 patients [61] (Table 1). The Australian Rheumatology Association and the National Institute for Health and Clinical Excellence (NICE) guidelines recommended that RA patients with long-term treatment with NSAIDs may continue their medication in the COVID-19 setting [29]. In contrast, the ACR recommended stopping NSAIDs in the cases of severe COVID manifestations [29]. The role of NSAIDs in the course of viral infections is still controversial. Recent preclinical data showed that although NSAIDs could suppress the inflammatory response in SARS-CoV-2 infection, they simultaneously impair humoral immune response against SARS-CoV-2 infection by dampening the production of protective antibodies [62]. In addition, ibuprofen and ketoprofen can aggravate the risk of secondary infections [63]. In contrast, some observations argued with the negative effects of ibuprofen and other NSAIDs in the COVID-19 setting and mentioned their prophylactic effects in COVID-19 [64,65]. Although ibuprofen can activate ACE2, which serves as a gateway for SARS-CoV-2 infection, it simultaneously endorses anti-inflammatory activity of Ras in the lungs and reduces the internalization of SARS-COV-2 spike protein by suppressing ADAM metallopeptidase domain 17 (ADAM17) and transmembrane protease serine 2 (TMPRSS2) activities [64,65]. The French Society of Pharmacology and Therapeutics recommended that the use of NSAIDs be avoided for symptomatic treatment in non-severe COVID-19 cases [65]. In a recent retrospective multi-centre observational study, non-selective COX inhibitors (aspirin and acetaminophen) were found to be associated with increased severity and mortality in COVID-19 patients including patients with pre-existing arthritis [66]. Fever is regarded as one of the indications of SARS-CoV-2 infection. Thus, the use of NSAIDs in the COVID-19 setting may delay in diagnosis of SARS-CoV-2 infection due to their antipyretic principle. However, treatment with selective COX-2 inhibitors (diclofenac, meloxicam, and celecoxib) was not found to be associated with an increase in COVID-19 severity [66]. Thus, it may be said that the effects of NSAIDs represent drug-specific risk profiles in the COVID-19 setting, and it is not worthy to mention their effects in a generalized way under a single class.

Hydroxychloroquine and chloroquine are commonly used as csDMARDs directed for the treatment of RA. Both these antimalarial drugs justify the theoretical requirements to be effective against COVID-19 [67]. Thus, these drugs were initially included in COVID-19 management. However, their effects remain controversial in COVID-19 management, and different sets of observations were reported in different clinical studies [1]. According to a previous report, these agents are not only ineffective in COVID-19 management but also possess the potential to cause more harm than benefit [29]. Patients chronically treated with hydroxychloroquine or chloroquine as anti-RA drugs before the COVID-19 pandemic did not exhibit any difference in COVID-19 outcomes compared to the patients who did not receive these antimalarial drugs [68]. Increasing evidence of COVID-19 death has been observed due to the cardiotoxic effect (arrhythmia) of these drugs used in the treatment of COVID-19 [25,59,69]. Chloroquine and hydroxychloroquine treatment have been found to develop QT prolongation in almost 10% of COVID-19 patients [1]. Thus, the WHO recommended against the use of hydroxychloroquine and chloroquine for COVID-19 treatment [70]. The ACR has also recommended a temporary suspension of hydroxychloroquine and chloroquine for RA patients in the setting of SARS-CoV-2 infection [25,59]. Similarly, the uses of other csDMARDs, such as leflunomide, methotrexate, and sulfasalazine have been advised to stop momentarily in RA patients during active SARS-CoV-2 infection (Table 1).

Considering the mechanistic similarity between RA and COVID-19 disease pathogenesis, it has been postulated that bDMARDs would be effective in the COVID-19 setting. However, the effect of bDMARDs in the COVID-19 setting remains highly controversial (Table 1). According to the National Health Service (NHS) England, RA patients treated with bDMARDs, such as tocilizumab, rituximab, and TNF inhibitors increase the risk of acquiring SARS-CoV-2 infection compared to the patients treated with csDMARDs [29]. In contrast, others were not in agreement with the claim of the NHS [25]. According to the data from the GRA global registry, out of 7263 COVID-19 positive cases among rheumatic patients, 2188 (30.13%) patients were under the treatment with bDMARDs [24]. Now coming to the continuation of bDMARDs in rheumatic patients in COVID-19 condition, anti-TNF therapy has been found to lower the disease severity and mortality [71]. GRA global registry data also claimed that using bDMARDs treatment (a largest subgroup of 52% RA patients used TNF inhibitors) by RA patients before acquiring SARS-CoV-2 infection significantly decreases the risk of hospitalization [24]. Anti-IL-6 (tocilizumab) treatment also had positive or equivocal results on COVID-19 outcomes [72]. In spite of the recommendation by some medical societies against the initiation or continuation of bDMARDs (including anti-TNF drugs) in the places where COVID-19 has been circulating in the community, the use of anti-IL-6 agents has been regarded to be safer [73]. The tendency of anti-TNF drugs to increase viral infection risk has been accounted for in this aspect. The risk of severe and opportunistic infections in RA patients under the treatment of tsDMARDs is roughly similar to that of bDMARDs in the COVID-19 setting [74]. Among tsDMARDs, janus kinase (JAK) inhibitors, such as baricitinib, tofacitinib, and upadacitinib, were studied in different trials [74]. Emerging evidence revealed that JAK inhibitors could possibly increase the risk of venous thromboembolism [75]. JAK inhibitors were also found to impair the IFN-mediated anti-viral response and to increase the risk of secondary infection [74]. Thus, medical associations recommended a temporary suspension of tsDMARDs to RA patients following COVID-19 exposure (Table 1). Precautionary withdrawal of DMARDs could simultaneously raise the chance of disease relapse and morbid outcomes in RA patients [76]. Thus, it would be a challenging task for the clinicians to treat RA patients after acquiring SARS-CoV-2 infection, who are maintained with DMARD drugs for RA management. However, the recommendations by different professional rheumatology associations, such as ACR and EULAR would serve as guidelines in this aspect. 

**Table 1:** Indications, contraindications, and recommendations of the important anti-rheumatic drugs used in COVID-19 setting.

Class of Drugs	Drugs	Indications	Risk Manifestations	Recommendations for RA Patients Acquiring COVID-19 Infection	References
**Glucocorticoids**	Dexamethasone, hydrocortisone, methylprednisolone.	Immunosuppressive agents and reduction in inflammation (during late phases of infection), mortality, and length of hospitalization.	Increase the risk of acquiring infection; a moderate-to-high dose can yield poor outcomes.	Continue at the lowest possible dose; however, sudden withdrawal is not recommended.	[23,25,59,69,77,78,79]
**NSAIDs**	Naproxen, celecoxib, etoricoxib, ibuprofen, ketoprofen, aspirin, acetaminophen.	Suppress inflammation and reduce fever.	Impair humoral immune response, increase the risk of bacterial infection, and increase severity and mortality (non-selective COX inhibitors).	Continue unless the patient with severe systemic manifestations.	[62,65,66]
**csDMARDs** Antimalarials	Hydroxychloroquine, chloroquine.	Not clearly understood, believed to exhibit antiviral effect via preventing viral entry, transport, and post-invasion events. Hydroxychloroquine is more potent and less toxic.	Dangerous when overdosed, cardiovascular side effect (QT prolongation). Maculopathy, retinal alteration, G6PD deficiency, and hypersensitivity are other contraindications. Special attention is required for injections.	Temporary suspension for RA patients with suspected/confirmed SARS-CoV-2 infection, patients with chronic heart failure, and/or patients receiving QT prolonging agents, such as azithromycin.	[25,67,69,80,81,82,83,84]
Other csDMARDs	Methotrexate, leflunomide, sulfasalazine.	Immunosuppressive agents; suppress inflammation.	Increase the risk of poor outcomes. Combination therapy yields poorer outputs then monotherapy.	Suspension for RA patients with suspected/confirmed SARS-CoV-2 infection.	[25,59,69]
**bDMARDs** Anti-TNF drugs	Adalimumab, infliximab, certolizumab pegol, etanercept, golimumab, secukinumab.	Suppress inflammation and reduce GM-CSF, VEGF, CRP, and blood coagulation.	Increase the risk of acquiring infection, hypersensitivity, and few cases of poor outcomes.	Suspension for RA patients with suspected/confirmed SARS-CoV-2 infection.	[25,29,69,71,84,85]
Anti-IL-1 drugs	Anakinra, canakinumab, rilonacept.	Suppress inflammation, prevent overpowering of innate immunity, improve oxygen saturation, reduce neutrophil counts, and inhibit Th17 cell induction.	Increase the risk of acquiring infection and hypersensitivity.	Suspension for RA patients with suspected/confirmed SARS-CoV-2 infection.	[25,69,84,86,87,88]
Anti-IL-6 drugs	Tocilizumab, sarilumab.	Suppress inflammation, prevent immune damage to target cells, improve oxygen saturation and reduce CRP, neutrophil counts, and fever.	Increase the risk of acquiring infection. Hypersensitivity, thrombocytopenia, leukopenia, aminotransferase elevation, and gastrointestinal perforations (rare) are other contraindications.	Initiation or continuation is recommended even in COVID-19-positive cases.	[68,84,89,90,91,92]
Anti-IL-17 drugs	Brodalumab, ixekizumab (LY2439821), secukinumab (AIN457).	Suppress inflammation; inhibit the production of IL-1, IL-8, and IL-6; exhibit immune-modulatory effect; and reduce neutrophil recruitment.	Increase the risk of acquiring infection.	Suspension for RA patients with suspected/confirmed SARS-CoV-2 infection.	[25,69,85,93]
Anti-IL-23 drugs	Guselkumab, risankizumab, tildrakizumab, ustekinumab.	Suppress inflammation, inhibit IL12/IL-23p40 or IL-23p19, and inhibit Th17 cell induction.	Increase the risk of acquiring infection and hypersensitivity.	Suspension for RA patients with suspected/confirmed SARS-CoV-2 infection.	[25,69,85,88]
**tsDMARDs** JAK inhibitors	Baricitinib, ruxolitinib, tofacitinib, upadacitinib.	Decrease virus infectivity, inhibit type-I/II cytokine receptors, reduce inflammation, and decrease neutrophil counts.	Impair IFN-mediated anti-viral response, increase the risk of secondary infection, venous thromboembolism, and hypersensitivity.	Suspension for RA patients with suspected/confirmed SARS-CoV-2 infection	[25,69,74,75,84,94]

bDMARDs: biological disease-modifying antirheumatic drugs; csDMARDs: conventional synthetic disease-modifying antirheumatic drugs; CRP: C reactive protein; GM-CSF: granulocyte-macrophage colony-stimulating factor; IL: interleukin; JAK: janus tyrosine kinase; NSAIDs: non-steroidal anti-inflammatory drugs; tsDMARDs: targeted synthetic disease-modifying antirheumatic drugs; Th cells: T helper cells; TNF: tumor necrosis factor; VEGF: vascular endothelial growth factor.

### 5.3. Monitoring RA Patients in the COVID-19 Setting

In the ongoing COVID-19 pandemic, the monitoring of RA patients is a significant challenge for rheumatologists. Patients with RA are known to be more susceptible to a multitude of infections, which advises against physically visiting the physicians on a routine basis during the COVID-19 setting. The COVID-19 lockdown also aids in this issue. Thus, in the absence of routine clinical assessment, it is really a challenging task for the rheumatologists to follow effective disease control protocol to alleviate disease activity. So, the situation forced rheumatologists to develop effective strategies to provide optimum patient care. Recent reports showed that telemedicine is gaining popularity in this situation [95]. It allows patients with stable disease conditions to reduce their visits to the clinic through adopting virtual consultation with rheumatologists. In India, rheumatologists have been preferring voice-over-IP service followed by video consultations and emails to provide virtual care [96]. 

Telephonic consultations supported by supplemental information, such as laboratory tests and photos of suspected manifestation areas, have also been found to be fruitful to avoid physical visits [97,98]. In this aspect, the RA impact of disease (RAID) score, a patient-derived score covering seven health areas: pain, fatigue, functional capacity, sleep quality, coping, physical well-being, and emotional well-being, has gained the reasonable confidence of rheumatologists for the virtual clinic to assess a composite measure of the disease activity [99]. Regarding flare assessment in the RA questionnaire, a self-reported flare can also serve as a potential tool in telehealth follow-ups with rheumatologists [100,101]. However, these teleconsultations do not allow proper clinical examinations, which are critical for a precise diagnosis. Since it is difficult to anticipate when the ongoing pandemic will end, rheumatologists must put in extra effort to build an appropriate telemedicine system that will ensure high-quality healthcare to RA patients [101]. In general, patients with stable disease activity could be advised to reduce their visits to clinics and to use virtual platforms for demonstrating disease activities, if required. However, patients who require frequent visits to the clinics for their disease severity and those who require other therapeutic requirements, or in case of emergency, could be advised to take all precautionary measures to avoid acquiring infection or to stop spreading the infection (for COVID-19-positive RA patients) [102].

## 6. Discussion

The clinical features of immune-inflammatory activities in SARS-CoV-2-infected alveolar structures share a pathological resemblance with the immunoreactive activities in the joints of RA patients. The establishment of localized acute inflammation mediated through excessive release of pro-inflammatory mediators is the hallmark of disease pathogenesis in both cases. Mechanistically, dysfunctional immune response endorses a hyperinflammatory state in alveolar tissue following SARS-CoV-2 infection referred to as a “cytokine storm.” In the cytokine-enriched environment, SARS-CoV-2 induces pyroptosis of lymphocytes, which results in the decline in memory Th cells and regulatory T lymphocytes. In addition, SARS-CoV-2 recruits APCs to trigger pathogenic Th17 cell activation, which induces neutrophilic inflammation and suppresses adaptive immune responses by activating neutrophils. In a “cytokine storm microenvironment,” CD4+ T cells recruit pathogenic Th1 cells, which aid in COVID-19 pathogenesis via macrophage activation. Immunologically, immune dysfunction against self”-antigens, as well as cytokine dysregulation, are hallmarks of RA. APC endorses Th2 and Th17 cell activation in the lymph node. CD4+ T cells in association with naive CD8+ T cells induce chronic autoimmune response and elicit inflammation via endorsing Th1/Th17 activation in synovial tissue. The activated Th1/IL-17 axis elicits the production of pro-inflammatory mediators, activates immune cells, and endorses B lymphocyte differentiation to produce autoantibodies in synovial tissue. In synovial fibroblasts, Th17 triggers the production of IL-17, which further promotes osteoclast development by recruiting pro-inflammatory cytokines, VEGF, RANKL, GM-CSF, and MMPs. Thus, the aberrant immune-inflammatory response resulting in hyperactivation of the cytokine–chemokine axis plays a crucial role in the pathogenesis of both COVID-19 and RA. With the emerging evidence that both RA and COVID-19 share similar pathological mechanisms in the affected tissues, it has been aimed to decipher the mechanistic overlapping between COVID-19 and RA immune-inflammatory features. Both COVID-19 and RA share common mechanistic pathways of pathogenesis mediated through aberrant ACE/ACE2 activities, as well as being driven by the activities of analogous macrophage clusters. The viral entry to the host cells depends on ACE2 binding of the spike protein of SARS-CoV-2 resulting in a suppression of ACE2 protein expression. Suppression of ACE2 and activation of ACE in alveolar tissues and joints share an identical mechanistic pathway of disease pathogenesis in both COVID-19 and RA, respectively. In addition, the alveolar macrophages in COVID-19 patients are homologous to that of the macrophages in joints of RA patients as seen with the macrophages in healthy alveolar tissue and joints, which strongly suggests that both COVID-19 and RA share similar mechanistic pathways of macrophage-mediated disease pathogenesis.

Towards effective therapeutic management, the use of anti-rheumatic drugs, such as glucocorticoids, NSAIDs, csDMARDs, bDMARDs, and tsDMARDs was critically reviewed along with their usefulness and contraindications in the COVID-19 setting. Although several anti-rheumatic drugs exhibited promise in certain clinical settings, careful investigation is obligatory to achieve their precise therapeutic utility in SARS-CoV-2-infected RA patients. Recognition of the immune-inflammatory mechanism of disease pathogenesis in both the diseases and the need for anti-rheumatic and anti-inflammatory drugs was largely underlined. Given the fact that several anti-rheumatic drugs do not influence COVID-19 severity/risk, RA patients may continue taking anti-rheumatic drugs during the pandemic. However, advice to RA patients on whether to stop or continue anti-rheumatic drugs requires careful testing of all disease-related parameters including severity, age, and comorbidities. In this line, we identified the immediate challenges and forwarded key recommendations by different professional rheumatology associations regarding the use of anti-rheumatic drugs in the COVID-19 setting for better clinical management of RA patients. Finally, routine monitoring of RA patients in the COVID-19 pandemic and the emerging prospects of telemedicine and virtual consultation were discussed.

## 7. Conclusions

Conclusively, COVID-19 and RA share similar immune-inflammatory features of disease pathogenesis executed by analogous mechanistic pathways. However, the treatment of RA patients in the COVID-19 setting itself stands as a challenging task. Implementation of individualized clinical surveillance of RA patients considering the disease severity and appropriate risk-benefit study referring to the recommendations of using anti-rheumatic drugs in the COVID-19 setting by different professional rheumatology associations would stand as the optimal therapeutic strategy for effective disease control during the COVID-19 pandemic. Finally, it is advisable to use virtual consultation by the patients with stable RA activity during this pandemic era, while the patients with RA flares are recommended to take all precautionary measures during visiting the clinics to prevent acquiring/spreading SARS-CoV-2 infection.

## Abbreviations

ACE	Angiotensin-converting enzyme
ACE2	Angiotensin-converting enzyme 2
ACPA	Anti-citrullinated peptide antibody
APC	Antigen presenting cell
bDMARDs	Biological disease-modifying antirheumatic drugs,
BMD	Bone marrow density
COVID-19	Coronavirus disease 2019
CRP	C reactive protein
csDMARDs	Conventional synthetic disease-modifying antirheumatic drugs
FABP	Fatty-acid-binding protein
FCN	Ficolin
FOLR	Folate receptor
GM-CSF	Granulocyte-macrophage colony-stimulating factor
IFN	Interferon
IL	Interleukin
JAK	Janus tyrosine kinase
LYVE	Lymphaticvessel hyaluronan receptor
NF-κB	Nuclear factor kappa-light-chain-enhancer of activated B cells
NSAID	Non-steroidal anti-inflammatory drug
RA	Rheumatoid arthritis
SARS-CoV-2	Severe acute respiratory syndrome coronavirus 2
Th cells	T helper cells
TNF	Tumor necrosis factor
tsDMARDs	Targeted synthetic disease-modifying anti-rheumatic drugs
VEGF	Vascular endothelial growth factor
